# BMP7 Gene involved in nonsyndromic orofacial clefts in Western han Chinese

**DOI:** 10.4317/medoral.20335

**Published:** 2015-02-07

**Authors:** Qiongqiong Yu, Sha He, Ni Zeng, Jian Ma, Bihe Zhang, Bing Shi, Zhonglin Jia

**Affiliations:** 1 State Key Laboratory of Oral Diseases, West China Hospital of Stomatology, Sichuan University, Chengdu, China; 2 Department of Cleft Lip and Palate Surgery, West China Hospital of Stomatology, Sichuan University, Chengdu, China

## Abstract

**Background:**

Nonsyndromic orofacial clefts (NSOCs) are the most common craniofacial birth defects with complex etiology in which multiple genes and environmental exposures are involved. Bone morphogenetic protein 7 (*BMP7*), as a member of the transforming growth factor-beta (TGF-beta) superfamily, has been shown to play crucial roles in palate and other orofacial ectodermal appendages development in animal models.

**Material and Methods:**

This study was designed to investigate the possible associations between *BMP7* gene and the NSOCs (221 case-parent trios) in Western Han Chinese. Five tagSNPs at *BMP7*, rs12438, rs6099486, rs6127973, rs230188 and rs6025469 were picked and tried to cover the entire gene. In order to identify the contribution of *BMP7* gene to the etiology of NSOCs, we performed several statistical analysis from different aspects including transmission disequilibrium test (TDT), pairwise linkage disequilibrium (LD), parent-of-origin effect and Chi-squared/Fisher’s exact tests.

**Results:**

Rs6127973 G allele and G/G homozygotes were over-transmitted for both NSOCs (*P*=0.005 and *P*=0.011, respectively) and NSCL/P (*P*=0.0061 and *P*=0.011, respectively), rs6127973 G allele was also paternally over-transmitted for both NSOCs (*P*=0.0061) and NSCL/P (*P*=0.011).

**Conclusions:**

This study suggested that rs6127973 may be a risk factor of being NSOCs and confirmed the role of *BMP7* gene in orofacial deformity from Western Han Chinese, which will also supply scientific evidence for future research and genetic counseling.

**Key words:** Single nucleotide polymorphisms, nonsyndromic orofacial clefts, BMP7.

## Introduction

Non syndromic orofacial clefts (NSOCs) are the most common craniofacial birth defects with the prevalence between 1/500-1/2000 worldwide (1,2). In general, Asians have the highest prevalence compared to the European and African population. The high incidence of orofacial clefts has significant physical and psycho social ramifications on the patients and their families (3).

With distinct etiologies and development patterns, non syndromic orofacial clefts are classified into two major phenotypes: non syndromic cleft lip with or without cleft palate (NSCL/P) and non syndromic cleft palate only (NSCPO). The 50% concordance rate of mono zygotic twins for cleft lip with or without cleft palate (CL/P) and cleft palate (CP) showed that both genetic and environmental exposures could alter susceptible risk of NSOCs, while the probandwise concordance rate was higher for CL/P and CP for mono zygotic twins than dizygotic twins, implied a strong genetic component, which promoted the discovery of the candidate genes (4-9).

Bone Morphogenetic Proteins (*BMPs*), a group of secreted signaling molecules of the TGF-beta super family (10), and their downstream targeting genes including Muscle segment homeobox1 (MSX1), are important regulators of craniofacial development. Experiments in chick led to the conclusion that both reduction and enhancement of *BMP* signaling within facial primordia caused defective lip fusion (11). Bone morphogenetic protein 4 (*BMP4*) deficient mouse displayed cleft lip (12), it is particularly important for lip development, as almost all conditional null embryos have bilateral cleft lip at embryonic day (E) 12, but less than 25% of embryos have cleft lip at E14.5 (12,13). The relationship between *BMP4* and lip development is further supported by mutations in individuals with micro form cleft lip (CL) and orbicularis oris defects (14).

*BMP7*, which locates on chromosome20q13, is a member of the 60A subfamily in the *BMP* family (15). *BMP7* predominately expressed in epithelium and mesenchyme of several orofacial structures including the edges of palatal shelves. *BMP7* deficient mice display cleft palate (16), and it has also been involved in the pathogenesis of human craniofacial malformations including NSCL/P (17). Mutations of *BMP7* in humans affect the development of teeth, palate and other orofacial complex (16,18,19). Besides, knockout mice for Msx1 and transforming growth factor-beta3 (TGF-beta3) genes, downstream targets of *BMP7*, exhibit cleft palate phenotype (19-21). Genetic studies have suggested that MSX1 mutations contributed to NSOCs in different populations (22-26). Thus we considered *BMP7* as a promising candidate gene for NSOCs and selected five SNPs (rs12438, rs6099486, rs6127973, rs230188 and rs6025469) in *BMP7* with minor allele frequency (MAF)>0.25 in Chinese Han Beijing (CHB) to identify the possible association between *BMP7* gene and NSOCs in Western Han Chinese.

## Material and Methods

- Study design and population 

The samples include 221 case-parent trios and 287 normal controls (without congenital malformation, having no family history of genetic disease, and matched by sex ratio as close as possible) ( Table 1), which were recruited between 2006 and 2013 from the Cleft Lip and Palate Surgery Department of West China Hospital of Stomatology, Sichuan University. Diagnosis of isolated NSOCs was determined through strict clinical genetic assessment. A brief interview was conducted with all participants’ mothers to gather environment factors including maternal vitamin supplementation (vitamin complex and folic acid) during the first trimester, maternal smoking history, maternal medication usage (antibiotics, cold cure, anti-emetics, analgesic, and anti-epileptic) during the first trimester and maternal abortion history. The research protocol was reviewed and approved by the local ethics committee. All participants were self-identified as Western Han Chinese.

**Table 1:**
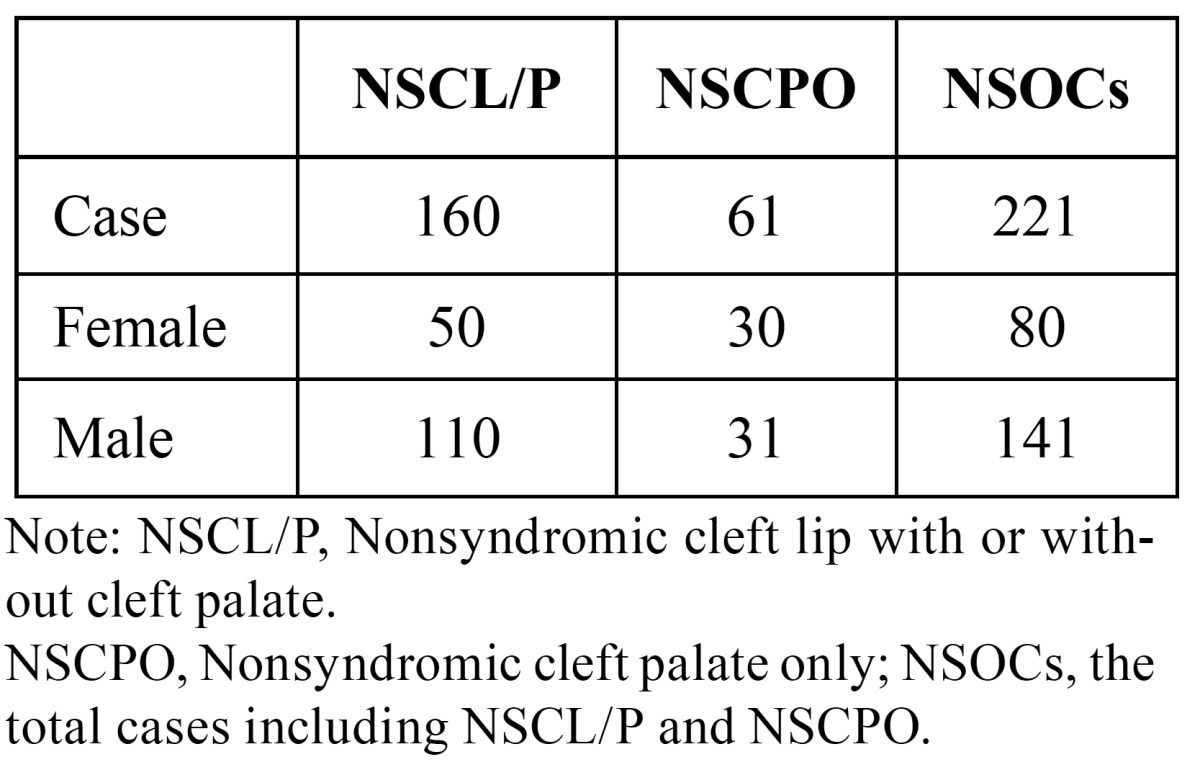
Gender and cleft type of cases.

- SNPs selection and genotyping

Peripheral venous blood samples (Neonatal umbilical cord blood for control individual) of all participants were drawn with their written informed consent. Genomic DNA was extracted using the protein precipitation method. We chose five tagSNPs (rs12438, rs6099486, rs6127973, rs230188 and rs6025469) from Hapmap CHB

 (http://hapmap.ncbi.nlm.nih gov/cgiperl/gbrowse/hapmap24_B36/#search) with the MAF>0.25 to obtain the maximum coverage of the *BMP7* gene. All the genotyping experiments were done by the Shanghai BioWing Applied Biotechnology Company (http://www.biowing.com.cn/) using ligase detection reactions (LDR). ( Table 2) shows the SNP information, Polymerase chain reaction (PCR) primers and probe sequences.

**Table 2:**
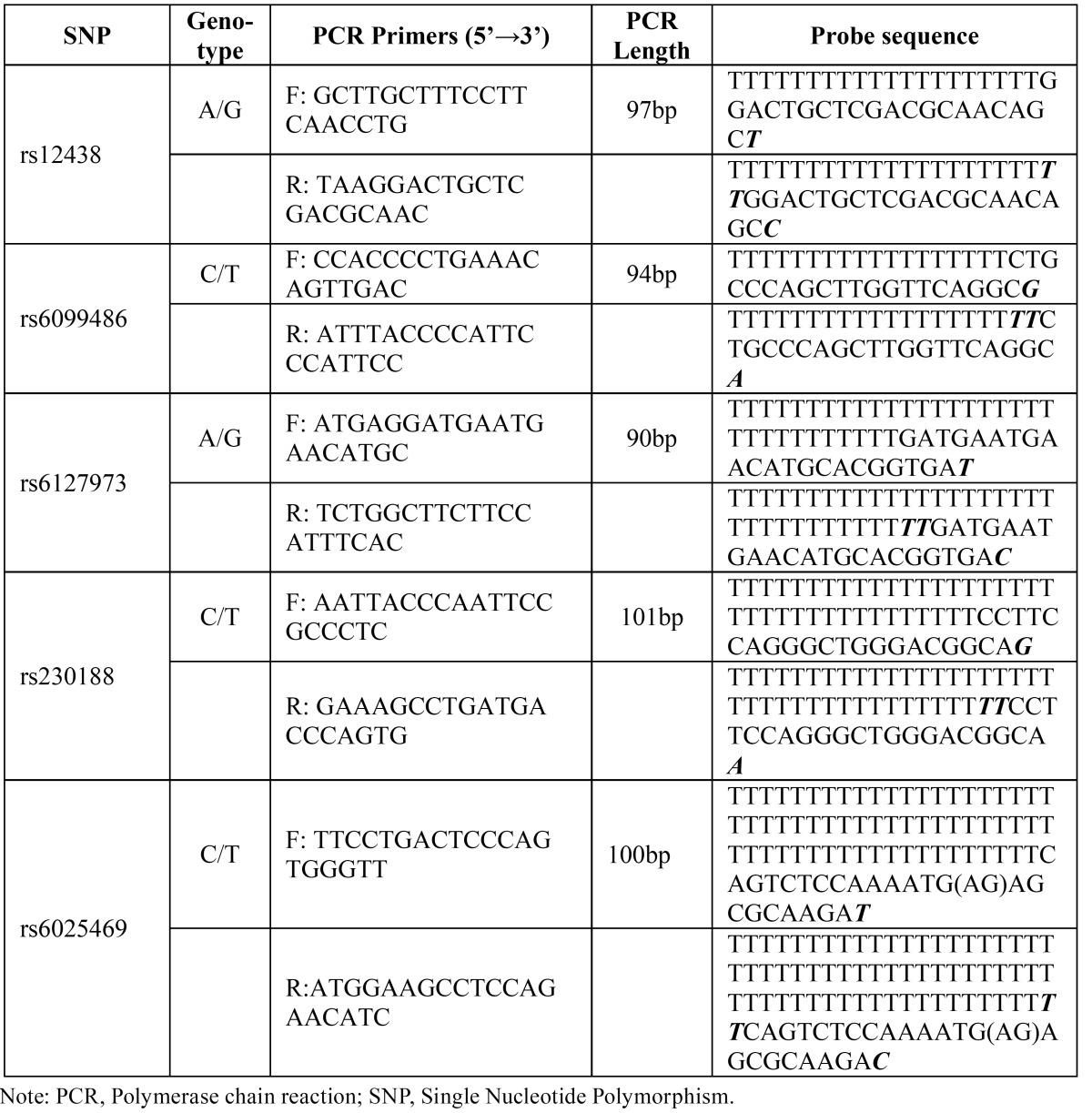
PCR primers and Probe sequence of the SNPs at BMP7.

- Statistical analysis

Hardy-Weinberg equilibrium (HWE) at each SNP was assessed among the normal parents. Pairwise linkage disequilibrium (LD) was computed as both D’ and r2 for all SNPs using the Haploview program.

(http://www.broad.mit.edu/haploview/haploview).

To identify LD blocks. Transmission disequilibrium test (TDT) were performed for individual SNPs to examine the transmission of target alleles and genotypes from heterozygous parents to the affected offspring by PLINK (27) and Family-based association test (FBAT) program.

(http://www.biostat.harvard.edu/~fbat/default.html). Parent-of-origin effect was assessed by PLINK to distinguish the parental preference of transmission on a disease variant. Gene-environment interaction was performed among case and control groups by Chi-squared/Fisher’s exact tests.

## Results

- Allelic and Genotypic TDT analysis

Significant deviation from Hardy-Weinberg expectations could reflect genotyping errors or true heterogeneity in the general population and could bias our statistical tests. Chi-squared tests using genotype frequencies among the normal parents and controls showed that the five SNPs were conformed to HWE.

Allelic TDT analyses from PLINK showed that A allele at rs6127973 was protective for NSOCs (*P*=0.0050, OR=0.66, 95%CI=0.50-0.89) and NSCL/P (*P*=0.0061, OR=0.62, 95%CI=0.44-0.87) ( Table 3). The allelic TDT did not show any significance between other SNPs and cleft group.

Genotype distribution comparison in TDT analyses form FBAT showed that G/G homozygotes at rs6127973, T/T homozygotes at rs6025469, A/G heterozygotes at rs12438 and C/T heterozygotes at rs6099486 were over-transmitted for NSOCs (*P*=0.011 and Z=2.54; *P*=0.035 and Z=2.11; *P*=0.0030 and Z=2.97; *P*=0.011 and Z=2.55, respectively) and NSCL/P (*P*= 0.011 and Z=2.54; *P*=0.032 and Z=2.14; *P*=0.016 and Z=2.40; *P*=0.035 and Z= 2.11, respectively) ( Table 4). TDT analyses also showed that C/T heterozygotes at rs6025469 and C/C homozygotes at rs6099486 were under-transmitted among NSOCs (*P*=0.047 and Z=-1.99; *P*=0.040 and Z=-2.05, respectively) and NSCL/P (*P*=0.028 and Z=-2.20; *P*=0.046 and Z=-2.00, respectively) ( Table 4). No evidence of association was identified in allelic or genotypic TDT analyses for NSCPO ( Table 3, Table 4). At rs230188, allelic and genotypic TDT both showed no evidence for association of NSOCs, NSCL/P or NSCPO.

- Parent-of-origin effects

Considering the parental origin of the alleles, there is no significant difference between the maternal and paternal among the SNPs for NSOCs, NSCL/P or NSCPO. However, we found an excess of paternal transmission of the allele G at rs6127973 for NSOCs (*P*=0.0061) and NSCL/P (*P*=0.011) ( Table 5). T allele at rs6025469 also displayed an excess of paternal transmission for NSOCs (*P*=0.029) ( Table 5). Other tests showed no evidence of paternal or maternal over/under-transmission ( Table 5).

- Interaction between *BMP7* polymorphism and environmental factors

We picked the significant associated SNP rs6127973 and the environmental factors (maternal vitamin supplementation during the first trimester, maternal smoking history, maternal medication usage during the first trimester and maternal abortion history) to do the gene-environmental interactions in NSOCs. However, we didn’t find any interaction between the risk genotypes and the environmental factors (data not shown).

**Table 3:**
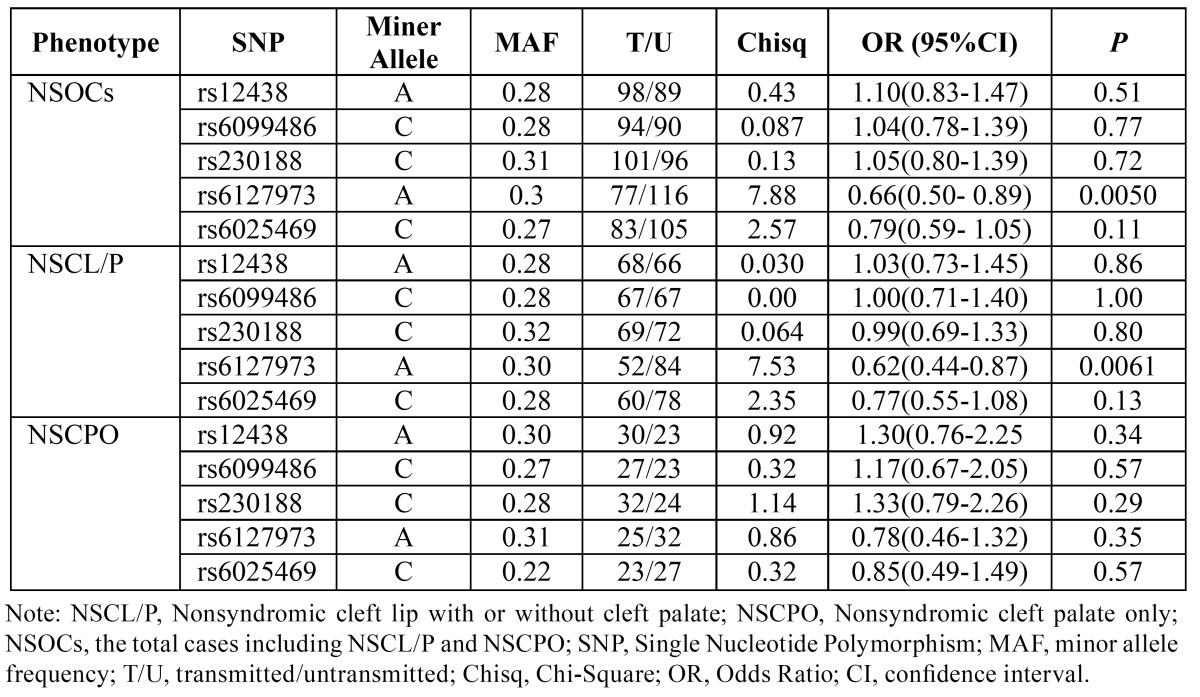
Allelic TDT results for SNPs in BMP7 from PLINK.

**Table 4:**
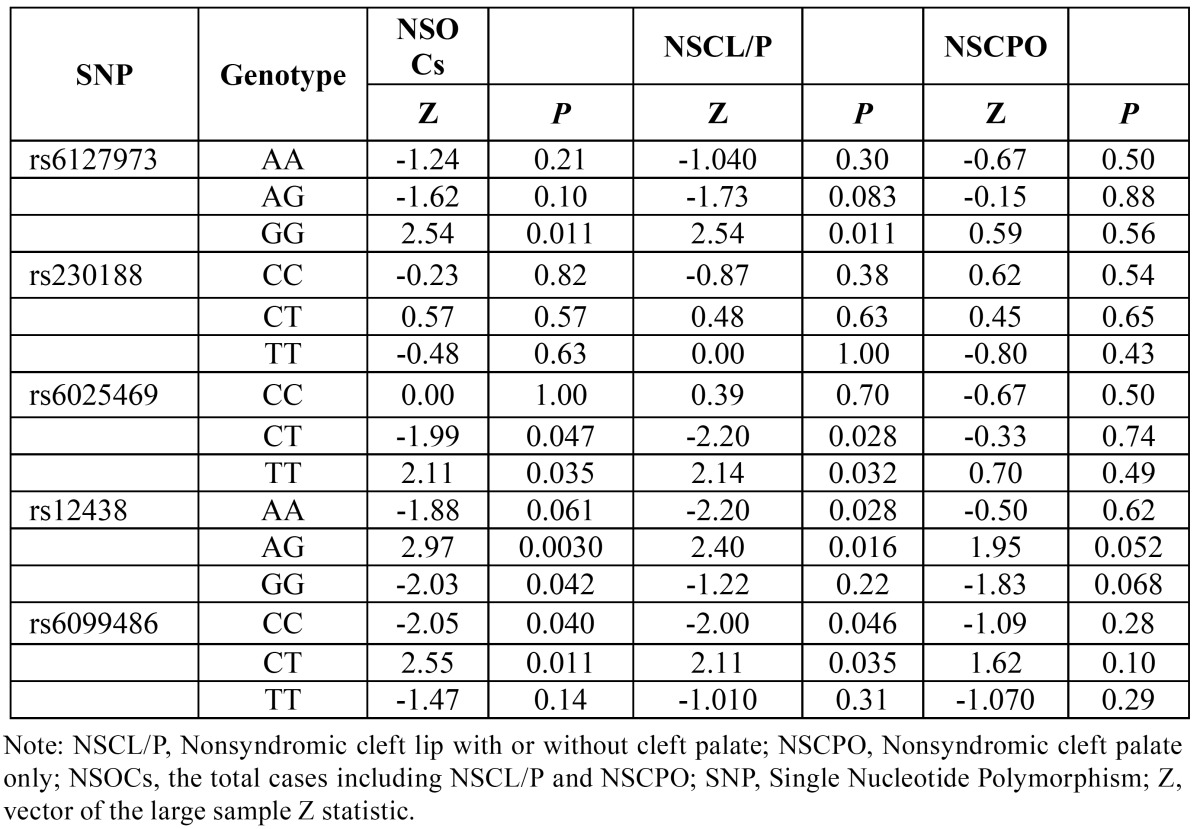
Genotypic TDT results for SNPs in BMP7 from FBAT.

**Table 5:**
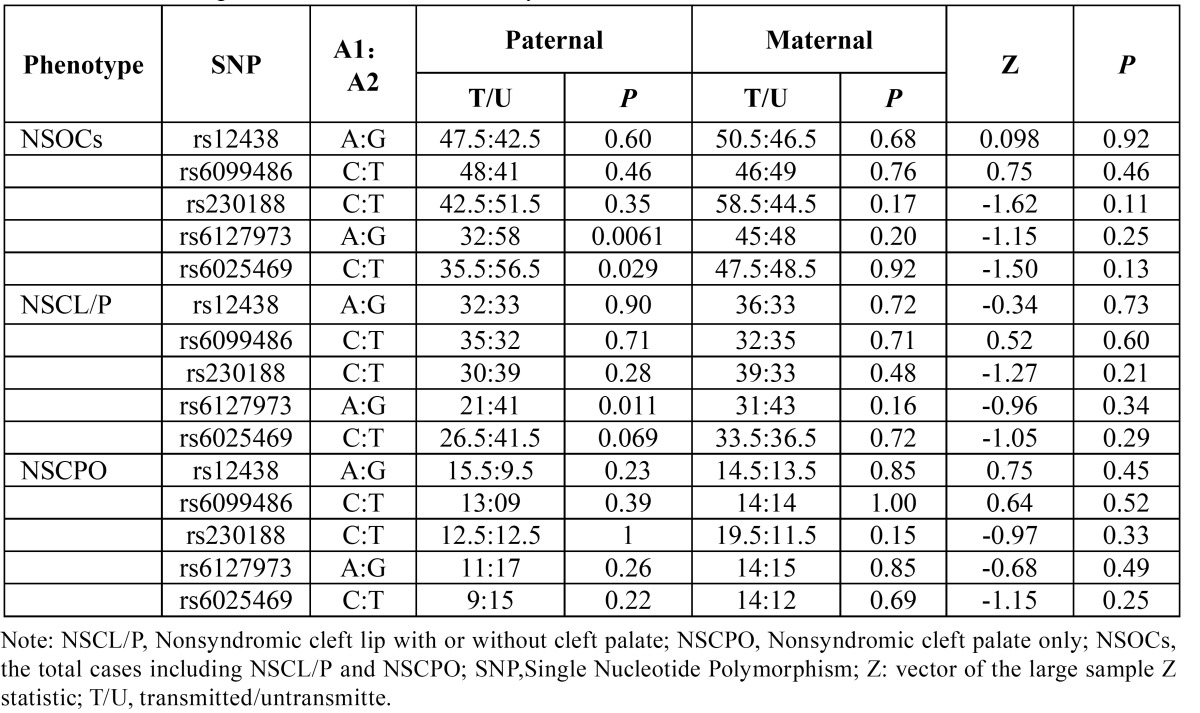
Parent-of-origin effects for SNPs in BMP7 by PLINK.

## Discussion

A multi factorial threshold model of inheritance with multiple, distinct causal genes are often assumed for NSCL/P (28), and there is no single gene model to explain the strong familial aggregation of NSCPO. The *BMP7* gene tested in the present study was expressed in the edges of palatal shelves. Deletions of *BMP7* in animal models have been proven its role in palate formation (18). Heterozygous variations in *BMP7* including a frame shift and missense mutation in individuals with a range of systemic abnormalities which included developmental delay, eye anomalies, deafness, scoliosis, and cleft palate further support that *BMP7* gene is associated with NSOCs (17).

As no study has been reported on the association between *BMP7* and NSOCs in Han Chinese, we picked five tagSNPs with the MAF>0.25 in CHB from Hapmap project and tried to cover the entire gene. The results showed that rs6127973 is associated with both NSOCs and NSCL/P, which is confirmed by both allelic TDT and genotypic TDT; we didn’t find significant associations between rs6025469, rs12438 and rs6099486 from allelic TDT analyses, but some genotypes were associated for both NSOCs and NSCL/P ( Table 3, Table 4).

To confirm if those five tagSNPs were independent with each other, we performed two-points LD analyses, the results showed very weak LD between the markers (D’<0.02, r2<0.70) as we assumed, which means that each of them could represents the other SNPs which was in the same LD block with it, and these tagSNPs could cover the entire BMP7 gene.

Parent-of-origin effects may occur when the phenotypic effect of an allele depends on whether it is inherited from an individual’s mother or father (29). In this study, it was taken into consideration with our family-based study design. The results showed no significant difference between the maternal and paternal. However, we did observe a paternally over-transmitted allele G on rs6127973 for NSOCs (*P*=0.0061) and NSCL/P (*P*=0.011) ( Table 5). A statistically significant transmission/disequilibrium test restricted to fathers but not mothers may be interpreted as evidence for non-expression of the maternally derived allele, which may reflect underlying imprinting (30). In general, epigenetic effects like imprinting are increasingly recognized as an important source of variation in complex traits (31). It must be emphasized the difference obtained for rs6025469 between the conventional TDT analysis and the parental stratified analysis. Considering the parental origin of the alleles, we observed a paternally over-transmitted allele T on rs6025469 for NSOCs (*P*=0.029), while allele T on rs6025469 showed no evidence of association for NSOCs in conventional allelic TDT analysis.

Though these loci resides in the 3’untranslated region and introns of the gene, many research literatures have recently suggested that 3’UTR and intron may contain some regulatory elements which have functions on gene transcription and translation efﬁciency, mRNA stability and polyadenylation signals (32-35).

Though located in intron, rs6127973 can alter four transcription factor binding sites, including activating transcription factor 3-known 9 (ATF3_known 9), activating transcription factor 6 (ATF6), X-box binding protein 1-1 (XBP-1-1) and p53-1 (Haploreg V2). Literatures have suggested that ATF6 may be involved in odontoblastic differentiation (36), which gave us a hint of the involvement of ATF6 in maxillofacial development. We will further detect this signal in animal model and identify the roles of ATF6 in maxillofacial development.

The etiology of NSOCs is recognized to be genetic or environmental risk factors. Epidemiological studies have suggested that genetic risk might interact with environmental agents (37-41), which play an important role in the etiology of NSOCs. However, our studies showed no significant correlation between the candidate gene and selected environment factors. The limited sample size is hard to detect the positive gene-environment interactions, stratifying into several subgroups also lead to smaller statistical power.

In aggregate, we confirmed the role of *BMP7* gene in orofacial deformity from Western Han Chinese, which will supply scientific evidence for future research and genetic counseling.
